# Unilateral *Exserohilum* Allergic Fungal Sinusitis in a Pediatric Host: Case Report

**DOI:** 10.1016/j.ijscr.2020.11.062

**Published:** 2020-11-16

**Authors:** Arwa A. Al Muslat, Basmah M. Alghmdi, Abdullah J. AlShehri, Rakan A. Alhaidey, M. Anas Dababo, Naif H. Alotaibi

**Affiliations:** a College of Medicine, Alfaisal University, Riyadh, Saudi Arabia; b Department of Surgery, College of Medicine, Alfaisal University, Riyadh, Saudi Arabia; c Department of Pathology and Laboratory Medicine, King Faisal Specialist Hospital, Riyadh, Saudi Arabia; d Department of Otolaryngology-Head & Neck Surgery, King Faisal Specialist Hospital and Research Center, Riyadh, Saudi Arabia

**Keywords:** Case report, *Exserohilum* species, Unilateral allergic fungal sinusitis

## Abstract

**Introduction:**

Allergic fungal sinusitis (AFS) is a result of an inflammatory reaction to fungi in the nasal and paranasal sinuses. Although the causative agents of AFS vary, *Exserohilum* species are among the rare ones, as only a few cases have ever been reported a few times in the literature. The objective of this report is to highlight this unusual fungal type causing unilateral AFS in Saudi Arabia.

**Presentation of the case:**

We present a case of AFS who initially presented at the age of 15 years, and was operated on in 2015 by another health care provider. He presented again in 2019 complaining of intermittent loss of smell and greenish nasal discharge, mainly from the right side for 3 months, which was associated with right-sided nasal obstruction. Based on the history and physical examination, a diagnosis of refractory chronic rhinosinusitis was made. The patient underwent functional endoscopic sinus surgery (FESS) of the paranasal sinuses and polypectomy. Histopathology of the samples taken during the surgery showed Exserohilum specie. The diagnosis of AFS was confirmed using Bent and Kuhn’s criteria.

**Discussion:**

Allergic fungal sinusitis is believed to be an allergic reaction caused by a broad spectrum of species, the majority being Aspergillus in India and Saudi Arabia. Exserohilum species are one of the rare organisms causing AFS in our region.

**Conclusion:**

Most cases of AFS in our region have been reported to be caused by Aspergillus. Here we report a case of unilateral *Exserohilum* AFS in an immunocompetent pediatric patient.

## Highlights

• Exserohilum species are a very rare causative organism in allergic fungal sinusitis (AFS).
• Treatment of AFS consists of medical and surgical modalities.
• AFS in pediatric patients is believed to be more aggressive with a higher recurrence rate.

## 1 Introduction

Allergic fungal sinusitis (AFS) is a result of an inflammatory reaction to fungi in the nasal and paranasal sinuses [1]. Patients with AFS usually present with chronic rhinosinusitis and nasal polyps that do not respond to conservative medical therapy [1], [2]. AFS can be distinguished clinically, histopathologically and by imaging from other chronic fungal sinusitis [2], [3]. The diagnostic criteria include type I hypersensitivity confirmed by history, skin test or serology, nasal polyposis, characteristic radiological findings, a positive fungal stain or culture and eosinophilic allergic mucin [3]. The mainstay of AFS treatment is surgical by functional endoscopic sinus surgery (FESS) along with medical treatment [4], [5], [6]. The literature reports a variety of causative agents but *Exserohilum* species are among the rare ones [7]. Although a few cases of AFS have been reported previously in our region, we present a case of unilateral AFS in a pediatric male patient due to a rare *Exserohilum* specie [8]. This case report has been written in line with the SCARE criteria [9].

## 2 Case report

**Fig. 1:**
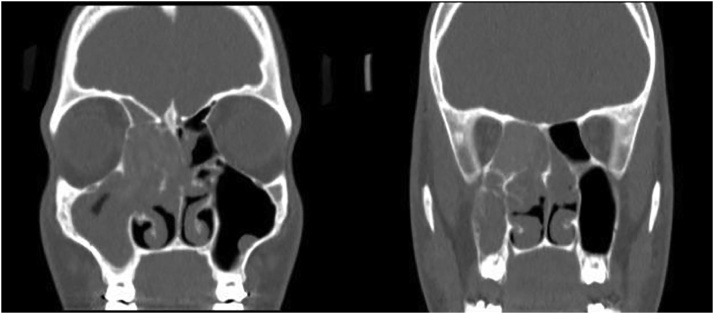
Preoperative imaging. In coronal view, the preoperative CT shows thick mucosal swelling outlining the antrum. Expanded ethmoid air cells are found on the right side. Radiopaque fungal elements are present within the opacified ethmoid air cells.

We present a case of AFS who initially presented at the age of 15 years and was previously operated on in 2015 by another health care provider. The patient presented to the Otolaryngology clinic at our institution (tertiary healthcare center) in 2019, complaining of intermittent smell loss and greenish nasal discharge mainly from the right side with acute intermittent nasal obstruction. Upon physical examination, smell diskettes test showed a result of 3/8 (anosmia), with an intact subjective retronasal smell (Novimed, Hemistrasse 46 CH-8953 Dietikon, Switzerland). Rhinoscopy showed second grade polyps based on Meltzer Clinical Scoring System in the right nasal cavity and a polypoid middle turbinate in the left cavity [10]. A computerized tomography (CT) scan of the paranasal sinuses showed thick mucosal swelling outlining the right maxillary sinus with expanded ethmoid sinus (Fig. 1).

**Fig. 2:**
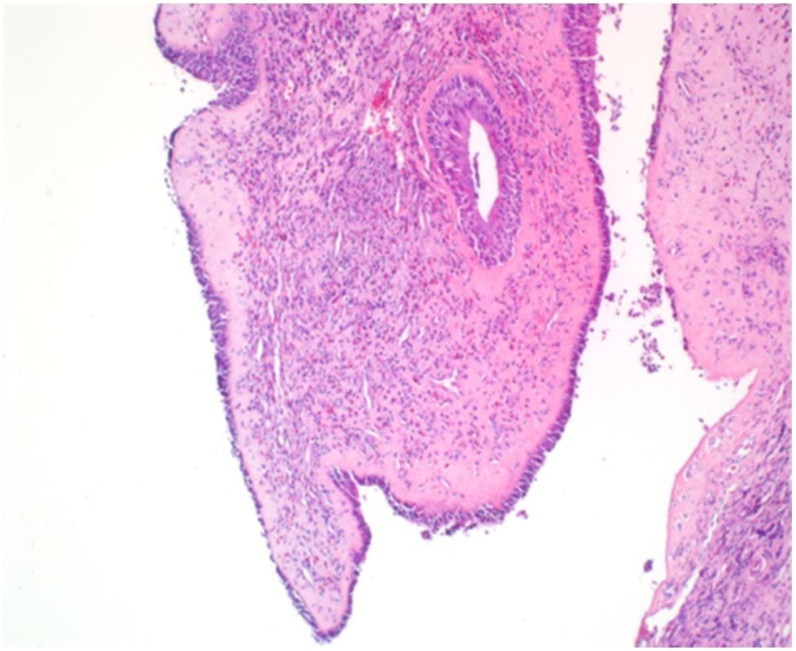
10× H&E showing polypoid mucosa with chronic inflammation mainly of eosinophilic infiltration.

**Fig. 3:**
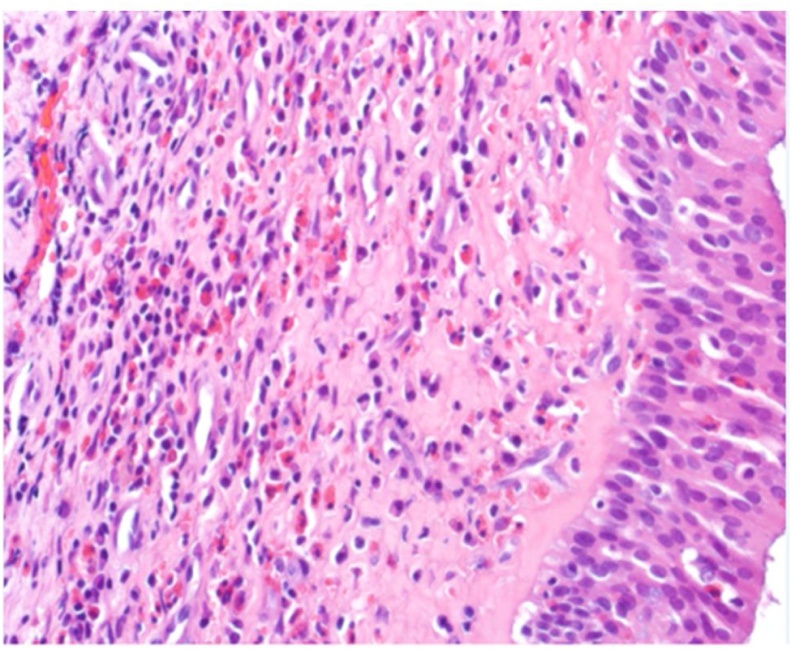
40× H&E showing the subepithelial marked eosinophilic infiltrate, with some eosinophils infiltrate the epithelial surface as well.

**Fig. 4:**
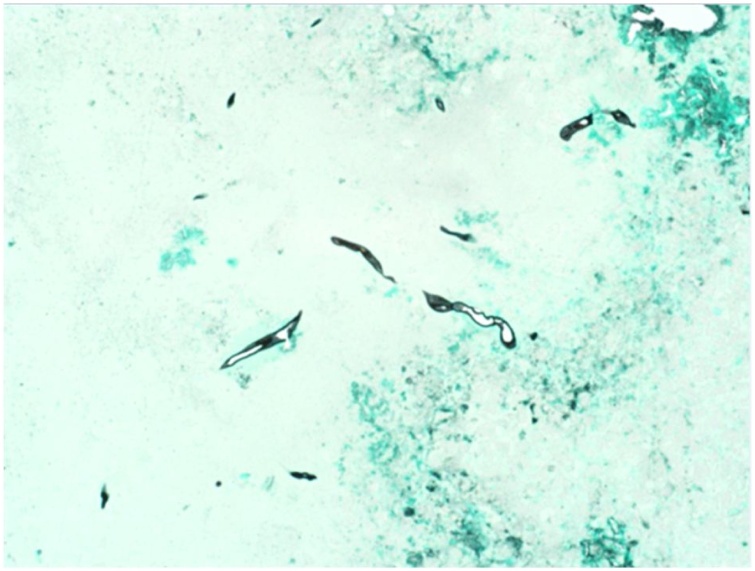
60× GMS special stain that shows the fungal elements within the allergic mucin (not in the tissue).

According to the history and physical examination a diagnosis of refractory chronic rhinosinusitis was made. The patient underwent revision FESS of the paranasal sinuses and polypectomy. Specimens taken during the surgery showed no invasive infection and the culture revealed *Exserohilum* species. Histopathology also reported the presence of eosinophilic infiltrate at the subepithelial and epithelial surfaces as well as the presence of fungal elements within the eosinophilic mucin (Fig. 2, Fig. 3, Fig. 4). The diagnosis of AFS was confirmed according to Bent and Kuhn's criteria [3]. No postoperative complications were reported, the patient was discharged and given a follow up appointment in 6 months. Upon follow up, the patient's signs and symptoms were reassuring and showed satisfactory outcomes. Written informed consent was obtained from the patient for publication of this case report and accompanying images. A copy of the written consent is available for review by the Editor-in-Chief of this journal on request.

## 3 Discussion

Allergic fungal sinusitis is believed to be an allergic reaction to aerosolized fungi present in the environment, in which a fungal allergen elicits a hypersensitivity reaction, affecting the paranasal sinuses and nasal cavity by fungal debris, allergic mucin and nasal polyposis. The fungi causing AFS have been identified on a broad spectrum of species. When examined histologically, most common cultures showed Dematiaceous fungi [1]. An epidemiological study showed the prevalence of dematiaceous fungi, especially Alternaria and Cladosporium spp. causing AFS in the United States. In contrast, 96.8% of AFS patients’ isolates in India showed *Aspergillus flavus* [11]. A retrospective review in Saudi Arabia was done on 45 pediatric patients, of which 25 of them met at least 4 of the diagnostic criteria of AFS. During their review, the most common fungus isolated was *Aspergillus* species [12]. As per previously reported specifically in India and Saudi Arabia, the majority of cases of AFS were caused by *Aspergillus flavus* [1].

We report a case of unilateral allergic fungal sinusitis by a rare type of fungi known as *Exserohilum* in an immunocompetent patient of a pediatric age group. AFS in pediatric patients was suggested to be more aggressive with a higher recurrence rate as compared to adults [13]. Of all mentioned causative organisms, infections caused by *Exserohilum* are rare. This type of species usually occurs in warm, tropical and subtropical areas such as the southern United States and India [14]. In a review from a single center in the southern United States, the most common fungi recovered from the paranasal sinuses were Bipolaris, followed by Curvularia and only a total of four patients had AFS caused by *Exserohilum*, two of which were children [15]. A case reported in Kuwait had a similar causative agent and age group as in our case. However, the finding in that case was bilateral in contrast to our case which is unilateral [8].

## 4 Conclusion

We encountered an unusual pediatric case of unilateral AFS caused by a rare *Exserohilum* species which to our knowledge has only been reported in a few cases [8]. Moreover, we aim to highlight the occurrence of AFS in an immunocompetent patient by an unusual organism which is unique to our region and the clinical spectrum of allergic fungal sinusitis [16].

## Declaration of Competing Interest

The authors report no declarations of interest.

## Sources of funding

None.

## Ethical approval

This study has been approved by research advisory counsel at King Faisal Specialist hospital in Riyadh Saudi Arabia (RAC #5166461).

## Consent

Written informed consent has been obtained from the patient, submitted and approved by the local IRB committee.

## Author contribution

Arwa Al muslat: First author, writing and editing – original draft, data collection and finalized the manuscript.

Basmah Alghmdi: First author, writing and editing – original draft, data collection and finalized the manuscript.

Abdullah J. Alshehri: Writing – review, editing and finalized the manuscript for submission.

Rakan Alhaidy: Participated in writing the discussion and literature review.

Muhammad A. Dabbabo: Contributed at managing the case, revised the manuscript.

Naif H. Alotaibi: The primary physician – treating and following up the patient, writing – supervision, critical revision of article and final approval for submission.

All authors approved the final version of the manuscript.

## Registration of research studies

King Faisal Specialist hospital and Research Center, Research ethics committee. RAC #5166461.

## Guarantor

Dr. Naif H. Alotaibi MD, Associated professor, College of Medicine, Alfaisal University, Department of Otolaryngology, King Faisal Specialist Hospital and Research Center, Riyadh, Saudi Arabia.

## Provenance and peer review

Not commissioned, externally peer-reviewed.
